# Glucose Metabolism in Children With Growth Hormone Deficiency

**DOI:** 10.3389/fendo.2018.00321

**Published:** 2018-06-11

**Authors:** Alessandro Ciresi, Carla Giordano

**Affiliations:** Biomedical Department of Internal and Specialist Medicine (DIBIMIS), Section of Endocrinology, University of Palermo, Palermo, Italy

**Keywords:** growth hormone, metabolism, glucose, children, insulin sensitivity

## Abstract

**Background:**

The growth hormone (GH)/insulin-like growth factor 1 (IGF-1) axis has a fundamental impact on glucose metabolism. Therefore, both untreated GH deficiency (GHD) and GH treatment (GHT) may be associated with some metabolic alterations, although the abnormalities of glucose metabolism have been investigated by relatively few studies as main outcomes.

**Aim:**

The present review summarizes the available data on glucose metabolism in children with GHD, providing an overview of the current state of the art in order to better clarify the real metabolic impact of GHD and GHT.

**Methods:**

Among all the existing studies, we evaluated all original studies that fulfilled our criteria for analysis reporting parameters of glucose metabolism as the primary or secondary objective.

**Results:**

The reported impact of GHD *per se* on glucose metabolism is quite homogeneous, with the majority of studies reporting no significant difference in metabolic parameters between GHD children and controls. Conversely, GHT proves to be more frequently associated with a subtle form of insulin resistance, while both fasting glucose and HbA1c levels remain almost always within the normal range.

**Conclusion:**

The different methods to study glucose metabolism, the heterogeneity of the populations evaluated, the different doses of GH used together with the variable duration of follow-up may be responsible for discrepancy in the results. Long-term longitudinal studies having glucose homeostasis as their primary outcome are still needed in order better to clarify the real metabolic impact of GHD and GHT in children.

## Introduction

The growth hormone (GH)/insulin-like growth factor 1 (IGF-1) axis has a fundamental impact on metabolism (1). GH regulates glucose homeostasis directly, by inducing glycogenolysis, gluconeogenesis, and lipolysis and promoting insulin resistance, and indirectly, *via* IGF-I production. Primarily, GH inhibits insulin-induced suppression of hepatic gluconeogenesis, thus increasing glucose production. In addition, it mainly act in stimulating lipolysis by providing free fatty acids (FFA) in order to switch metabolism from glucose and protein to lipid utilization (2–5) and these evidences were supported by a study that demonstrated that inhibition of lipolysis with acipimox, a free fatty acid blocker, partially prevented GH-induced insulin resistance (6).

Following the reduction of insulin sensitivity, a compensatory increase in insulin secretion is usually observed. In addition, GH is also a potent growth factor for β-cell proliferation and insulin secretion (7). Other mechanisms play a role in the metabolic effects of GH, namely its interaction with the insulin receptor (8, 9) and the presence of a polymorphism in the GH receptor gene (10–15). On the other hand, IGF-1 physiologically improves glucose homeostasis and enhances insulin sensitivity (16, 17). For these reasons, the interplay between GH/IGF-1 axis and glucose metabolism is complex.

Both untreated GH deficiency (GHD) and GH treatment (GHT) may be associated with metabolic alterations and a common mechanism could be the increased flux of FFA caused by body composition alterations (in untreated GHD) or enhanced lipid oxidation secondary to the anti-insulin effect of GH (during GHT), respectively (5, 18).

However, metabolic abnormalities associated with GHD have so far been evaluated only in a small number of children (19) and most studies focused on the main markers of cardiovascular disease, namely abnormalities in body composition (20–22), lipid profile (23, 24), inflammatory markers (25–27), and cardiac function (28–32), while the abnormalities of glucose metabolism have been investigated by relatively few studies and incompletely. The present review summarizes the available data on glucose metabolism in children with GHD, providing an overview of the current state of the art.

## Selection of Studies for Review

PubMed was searched for studies evaluating the glucose metabolism in children with GHD and/or its changes during GHT. The search terms used were “growth hormone deficiency in children,” “growth hormone deficiency and glucose,” “growth hormone deficiency and diabetes,” and “growth hormone deficiency and metabolism.” We excluded from the analysis the review articles. We considered all original studies in English language published from 1970 to January 2018 which reported as the primary or secondary objective of the study at least one of the following metabolic parameters: fasting glucose, fasting insulin, glycosylated hemoglobin (HbA1c), homeostatic model assessment of insulin resistance (Homa-IR), the quantitative insulin sensitivity check index (QUICKI), the insulin sensitivity index (ISI), or the *M*-value (derived from euglycemic hyperinsulinemic clamp).

Specifically, we carefully evaluated more than 100 studies concerning children who have been treated with GH. We considered case–control prospective studies performed on consecutive patients (levels of evidence I/II). We excluded from the analysis all original studies which evaluated less than five patients, those with a follow-up of less than 1 month of treatment, studies on children treated with long-acting formulations of GH or treated with GH for indications other than GHD. The final analysis comprised 22 (for naïve GHD children compared to controls) and 24 studies (for children during GHT), that fulfilled our criteria for analysis.

## Results

### Glucose Metabolism in Children With GH Deficiency

Low GH and IGF-1 levels are known to be predictors of unfavorable metabolic profile in healthy subjects (33–35) and in adults with GHD (36–39). Similarly, adolescents with confirmed GHD who discontinued GHT at completion of linear growth may exhibit metabolic alterations (23, 25, 40–48), as well as untreated GHD in children has also been associated with many markers of cardiovascular risk (9, 49, 50).

Substantially, most of the studies did not show a significant impairment in glucose metabolism in naïve GHD children. The Table 1 summarizes the studies on glucose metabolism in naïve GHD children compared to controls.

**Table 1:** Selected studies for analysis of glucose metabolism in naïve GHD children compared to controls.

Reference	No of patients	Gender (M/F)	Fasting glucose	Fasting insulin	HbA1c	Homeostatic model assessment of insulin resistance	Quantitative insulin sensitivity check index	Insulin sensitivity index	*M*-value
Lippe et al. (52)	11	–	↔	↓	–	–	–	–	–
Heptulla et al. (70)	8	4/4	↔	↔	–	–	–	–	–
Seminara et al. (66)	20	15/5	↔	↔	↔	–	–	–	–
Husbands et al. (53)	91	–	↓	–	–	–	–	↑^a^	–
Radetti et al. (57)	128	88/40	–	–	–	–	↓	–	–
Salerno et al. (30)	30	18/12	↔	↔	–	↔	–	–	–
Ciresi et al. (58)	34	25/9	↔	↔	–	↔	–	–	–
Lòpez-Siguero et al. (27)	30	19/11	↔	↔	–	↔	–	–	–
Canete et al. (26)	36	22/14	↓	↓	–	↓	–	–	–
Metwalley et al. (60)	30	18/12	↔	↔	–	↔	–	–	–
Prodam et al. (13)	23	–	↔	↔	–	↔	–	–	–
Meazza et al. (67)	16	11/5	↔	–	–	–	–	–	–
De Marco et al. (61)	20	6/14	↔	↔	↔	↔	–	–	–
Ramistella et al. (59)	32	17/15	↔	↔	–	↔	–	–	–
Ciresi et al. (62)	73	55/18	↔	↔	↔	↔	↔	↔	–
Ciresi et al. (65)	31	25/6	↔	↔	↔	↔	↔	↔	–
Ciresi et al. (63)	48	32/16	↔	↔	↔	↔	↔	↔	–
Stawerska et al. (54)	26	15/11	↔	↔	–	↔	–	–	–
Ciresi et al. (77)	23	15/8	↔	↔	↔	–	–	↔	↔
Ciresi et al. (64)	51	35/16	↔	↔	↔	↔	–	↔	–
Capalbo et al. (68)	100	60/40	↔	↔	↔	↔	–	–	–
Chen et al. (69)	60	37/23	↔	–	–	–	–	–	–

*GHD, growth hormone deficiency*.

*↔, comparable with controls; ↑, higher than controls; ↓, lower than controls; –, not available*.

*^a^Insulin sensitivity was assessed by the rate of glucose disappearance during an insulin tolerance test*.

Fasting hypoglycemia and marked insulin sensitivity have sometimes been observed in GHD children due to diminished hepatic output through decreased gluconeogenesis or abnormal glucose mobilization (51). One of the first metabolic studies performed in 11 GHD children has shown basal hypoinsulinemia with normal fasting glucose compared with controls (52). Partially similar results were documented by Canete et al. in 36 GHD children, who showed lower glucose, insulin, and Homa-IR values than controls (26). The tendency of GHD children to show higher insulin sensitivity has been demonstrated by Husbands et al. (53). In that study, as a direct measure of insulin sensitivity the authors evaluated the rate of glucose disappearance following an insulin tolerance test in 91 GHD children and 142 controls. The authors demonstrated that GHD children had an increased susceptibility to hypoglycemia, thus proving to be more insulin-sensitive than GH-sufficient children.

Partially in accordance with these data, more recently Stawerska et al. demonstrated comparable glucose and insulin levels between GHD children and controls but significantly lower insulin levels and higher insulin sensitivity in GHD children with lower IGF-1 bioavailability than children with higher IGF-1 levels (54).

The insulin sensitivity related to GHD seems to decrease with age, probably due to the physiological effect of steroids production or the modifications of body composition during the time. Indeed, in normal children insulin action is physiologically reduced during puberty and insulin secretion is normally increased. The insulin resistance of puberty, which was found to be directly correlated with IGF-1 levels, leads to compensatory hyperinsulinemia which serves to amplify the anabolic effect of insulin during this period of rapid growth (55, 56). For these reasons, the higher insulin sensitivity observed in young untreated GHD children is widely different from the different degree of insulin resistance shown by adolescents (19) or adults (39) with GHD, in which the change of body composition plays an additional fundamental role (43, 44).

These data contrast with those of Radetti et al., who through evaluation of QUICKI demonstrated a slight degree of insulin resistance in GHD children at baseline compared with controls (57), while other studies did not show any impairment in Homa-IR (12, 27, 30, 58–61) or in ISI (38, 62–65) in naïve children. No significant difference in fasting glucose, insulin, and HbA1c levels between GHD children and controls were found in many other studies (38, 61–70).

### Glucose Metabolism During GH Treatment

Growth hormone treatment has been suggested to impair glucose metabolism because of the anti-insulin effect of GH and its direct effect on β-cell, although the real metabolic impact of GHT in children has not been established. The limitation of most studies is represented by evaluation of glucose metabolism through basal parameters and surrogate indices of insulin sensitivity, i.e., fasting glucose, fasting insulin, or Homa-IR, and not by the more reliable indexes derived from oral glucose tolerance test (OGTT), i.e., ISI Matsuda, or the gold standard technique to measure insulin sensitivity, the euglycemic hyperinsulinemic clamp (71), which is expensive and not routinely applicable.

The Table 2 summarizes the studies on glucose metabolism in GHD children during GHT.

**Table 2:** Selected studies for analysis of glucose metabolism in GHD children during GHT.

Reference	No of patients	Gender (M/F)	Mean follow-up (months)	Fasting glucose	Fasting insulin	HbA1c	Homeostatic model assessment of insulin resistance	Quantitative insulin sensitivity check index	Insulin sensitivity index	*M*-value
Lippe et al. (52)	11	–	6	↔	↔	–	–	–	–	–
Heptulla et al. (70)	8	4/4	6	↔	↑	↔	–	–	–	–
Seminara et al. (66)	20	15/5	36	↔	↑	↔	–	–	–	–
Radetti et al. (57)	128	88/40	72	–	–	–	–	↓	–	–
Salerno et al. (30)	30	18/12	24	↔	↑	–	↑	–	–	–
Nozue et al. (75)	20	16/4	1	↔	–	–	–	–	–	–
Ciresi et al. (58)	34	25/9	12	↔	↑	–	↑	–	–	–
Lòpez-Siguero et al. (27)	30	19/11	12	↔	↔	–	↑	–	–	–
Canete et al. (26)	36	22/14	6	↔	↑	–	↑	–	–	–
Metwalley et al. (60)	30	18/12	12	↑	↑	–	↑	–	–	–
Meazza et al. (67)	16	11/5	12	↔	↑	–	↔	–	–	–
De Marco et al. (61)	20	6/14	12	↔	↔	↔	↔	–	–	–
Ramistella et al. (59)	32	17/15	24	↔	↑	–	↑	–	–	–
Ciresi et al. (62)	73	55/18	12	↑	↑	↑	↑	↓	↔	↓
Wei et al. (76)	45	30/15	12	↔	–	–	–	–	–	–
Baronio et al. (81)	99	62/37	72	–	–	–	↑	–	–	–
Ciresi et al. (65)	31	25/6	12	↔	↑	↔	↑	–	–	–
Ciresi et al. (63)	48	32/16	24	↑	↑	↔	↑	↓	↓	–
Xue et al (72)	60	39/21	6	↑	–	–	–	–	–	–
Ciresi et al. (77)	23	15/8	12	↔	↑	↔	–	–	↓	↓
Ciresi et al. (64)	51	35/16	12	↑	↑	↔	↑	–	↓	–
Ciresi et al. (80)	32	25/7	12	↑/↔^a^	↑	↔	↑	–	↓/↔^b^	–
Capalbo et al (68)	100	60/40	60	–	↑	–	↑	–	–	–
Chen et al (69)	60	37/23	12	↑	–	–	–	–	–	–

*GHD, growth hormone deficiency; GHT, growth hormone treatment*.

*↔, unchanged; ↑, increased; ↓, decreased; –, not available*.

*^a^Increased with daily injection of GH, unchanged with three injections per week*.

*^b^Decreased with daily injection of GH, unchanged with three injections per week*.

Fasting glucose is undoubtedly the parameter mostly used to evaluate glucose metabolism in almost all studies (21/24; 87.5%). In a recent study, Xue et al. reported a significant increase in fasting glucose in 60 children during 6 months of GHT, although in half of the patients a high GH dose (0.1 U/kg) was used (72). Similar results have been shown by Chen et al. in 60 children after 12 months of GHT, regardless of the GH dose used (68) and these data have been confirmed by four other studies (60, 62–64).

In 2000, a large retrospective analysis of data more than 23,000 children reported that the incidence of type 1 diabetes did not differ from expected values, while the incidence of type 2 diabetes was higher (85 out of 23,333 children, 0.36%) than reported in children not GH-treated, probably as a consequence of an acceleration of the disorder in predisposed individuals. However, it has to be highlighted that not only children with GHD were included in this analysis, but also children with other clinical conditions treated with supraphysiological doses of GH (73).

In 2011 an analysis of data of 11,686 patients showed a slightly higher incidence of type 2 diabetes in GH-treated children than in the general population. Interestingly, most patients who developed diabetes had preexisting risk factors, like preexisting insulin resistance. Thus, monitoring of glucose before and periodically during GHT is recommended by the authors, especially for children with preexisting risk factors (74).

Radetti et al. reported normalization of glucose tolerance in 5 out of 128 children who already presented impaired glucose tolerance before starting GHT (57), while unchanged glucose levels have been found in the majority of studies (14/21; 66.6%) (12, 15, 26, 52, 58, 59, 61, 65, 66, 70, 75–77).

Recently, a large safety-monitoring study enrolling more than 54,000 children who were given GHT and were followed until GHT discontinuation showed no significant increase in incidence of diabetes compared to the general population (78), as well as a recent analysis failed to show an increased incidence of diabetes in adults who were previously treated during childhood (79). Notably, a recent study by our group reported a significant increase in fasting glucose only in children who were given GHT every day compared to those who were given the same dose through three injections per week (80). The limit of the use of fasting glucose as a parameter to detect changes in glucose metabolism during GHT has been documented by Lippe et al., who demonstrated higher plasma glucose concentrations during OGTT than controls after 6 months of GHT, despite mean fasting glucose remaining normal (52).

HbA1c was only evaluated in 9 out of 24 studies and most of these (8 studies) showed no significant change in levels during GHT.

Another metabolic parameter very frequently assessed during GHT is fasting insulin, evaluated in 18 out of 24 studies (75%). An increase in fasting insulin has been documented in 83.3% of studies and unchanged levels in 11.1% of studies. Lippe et al. showed comparable fasting insulin levels between GHD children and controls but with a concomitant increase in insulin secretion following OGTT in a group of 11 children after 6 months of GHT (52).

Insulin sensitivity has been more frequently studied by surrogate indexes derived from fasting glucose and insulin levels, as Homa-IR or, less frequently, QUICKI.

Homeostatic model assessment of insulin resistance was calculated in 15 out of 24 studies. Specifically, it proved to be unchanged in two studies (13%) (61, 66) and increased in the majority of them (87%). An increase in fasting insulin levels and, consequently, in Homa-IR after 2 years of GHT was documented by Salerno et al. (30). Similarly, López-Siguero et al. showed a significant increase in Homa-IR, without significant change in fasting glucose and insulin levels, in 30 GHD children after 12 months of GHT (27). Conversely, Metwalley et al. documented a deterioration in Homa-IR with a concomitant increase in fasting glucose during GHT (60), while Meazza et al. documented a rise in fasting insulin but without changes in Homa-IR (67). Ramistella et al. confirmed an increase in both fasting insulin and Homa-IR in 32 children, although these values were within the normal range during the entire follow-up (59) and a similar deterioration in insulin sensitivity assessed by Homa-IR was reported by other authors (26, 58, 62–64, 68).

Quantitative insulin sensitivity check index was evaluated in very few studies (4 out of 24 studies; 16.6%) and it is proved to decrease during GHT in all of them (57, 62, 63, 65).

Fewer studies evaluated the degree of insulin sensitivity through evaluation of the more reliable ISI (6 out of 24; 25%) and the majority of them (5 out of 6; 83%) are concordant in detecting a reduction in ISI during GHT (63–65, 80).

The clamp was used in very few studies. Heptulla et al. employed the hyperglycemic clamp to evaluate the effects of 6 months of GHT in six children affected by GHD and two children with non-deficient short stature. The authors showed a decrease in insulin sensitivity compensated by a marked increase in insulin responses after GHT, although it remained within the normal range (70). The few studies that directly assessed insulin sensitivity by euglycemic hyperinsulinemic clamp showed a significant reduction in *M*-value, hence confirming a real deterioration in insulin sensitivity after GHT, though without evident changes in glucose tolerance (62, 77).

Recently, 99 GHD children were annually tested with OGTT to longitudinally study insulin sensitivity and the capacity of β-cells to adapt to changes in insulin sensitivity (through the oral disposition index, DIo) during 6 years of GHT. The results of this study suggested a positive influence of GHT on the β-cell secretory capacity, without a significant impact on insulin sensitivity (81). The DIo was also evaluated in another study enrolling 73 GHD children, who showed a significant reduction in DIo in concomitance with an increase in insulin levels during GHT. The conclusion of the study was that while a direct trophic effect of GH on β-cells cannot be ruled out to explain the increase in fasting insulin secretion, the decrease in DIo can be considered an early marker of inadequate β-cell compensation of decreased insulin sensitivity (62).

It is important to highlight that, overall, the anti-insulin effect of GH seems to be prevalent during the first months of treatment and may be caused by a decline of peripheral glucose utilization and increase in insulin resistance (82) whereas, after initial deterioration, glucose tolerance can improve (83). Capalbo et al. demonstrated that the first year of GHT was associated with an increase in insulin and HOMA-IR, but these parameters did not change further during the following years of treatment. Indeed, at the fifth year of the study a significant increase in insulin and HOMA-IR was documented also in control subjects, making these parameters comparable between GHD and controls (68). This finding is consistent with the results of Radetti et al., who showed a slight deterioration in insulin sensitivity after the first year of GHT, but without a further progressive deterioration in the following 6 years (57).

In synthesis, glycometabolic effects of GHT seem to be biphasic, leading initially to deterioration of glucose metabolism, but in the long-term an improvement of glucose handling is reported. These data can be explained by the long-term beneficial effects of GH on body composition, which probably may overcome the insulin-antagonist effect of GH in the long-term follow-up (84).

### Predictors of Glucose Alterations

Few studies have tried to identify any predictive factors of glucose alterations in children during GHT. Probably, the strongest predictors of the degree of insulin resistance and the development of diabetes during GHT are represented by the individual predisposition of patients or by the presence of preexisting diabetes risk factors (73, 74). Interestingly, 1 h-glucose levels higher than 132.5 mg/dl after OGTT at diagnosis were found to be a predictor of alterations in glucose metabolism during GHT (64), in agreement with other studies performed in non-GHD children (85). Recently, Staverska et al. demonstrated that GHD children are a heterogeneous group as regards the differences in the metabolic profile, probably due to the different IGF-1 bioavailability, and concluded that naïve GHD children have a worse metabolic profile when the IGF-1 bioavailability and the body mass were greater, although the authors do not fully explain this phenomenon (54). In addition, the role of microRNAs expression in skeletal muscles has been hypothesized as a mechanism that contributes to increased insulin resistance during GHT (86).

## Concluding Remarks

Glucose metabolism in GHD children is not exhaustively and uniformly evaluated by the available studies. Fasting glucose and insulin are the parameters most frequently evaluated, followed by Homa-IR and HbA1c levels. Very few studies used other metabolic parameters, i.e., QUICKI, ISI, and *M*-value.

In children, the reported impact of GHD *per se* on glucose metabolism is quite homogeneous, with the majority of studies reporting no significant difference in metabolic parameters between GHD children and controls. Conversely, GHT proves to be more frequently associated with a subtle form of insulin resistance that can be assumed to be the mechanism by which basal glucose does not significantly change after GHT despite the increase in insulin levels.

The increase in insulin levels and, consequently, in Homa-IR during GHT probably does not indicate with any certainty a condition of insulin resistance, probably due to the inability of basal indexes to reliably assess insulin sensitivity. Indeed, the increase in Homa-IR, as well as the reduction in QUICKI, may just represent an expected consequence of GH-induced basal hyperinsulinemia and very few studies have compared data about the degree of insulin sensitivity in GHD children assessed by different indices (77). However, even in the few studies that used more reliable indices, like ISI or *M*-value during clamp, a reduction in insulin sensitivity during GHT was almost always confirmed.

Overall, despite the decrease in insulin sensitivity, both fasting glucose and HbA1c levels remain almost always within the normal range. Therefore, the hyperinsulinemia documented during GHT can probably be compared to that observed during puberty (87), although the long-term implications of this prolonged state of hyperinsulinemia are not widely known. Probably, the long-term beneficial effect of GHT on body composition will counterbalance the initial insulin-antagonist effects of GH leading to a general improvement in insulin sensitivity.

For these reasons, to date there are no clear indications on routinely monitoring GHD patients receiving GHT with regards to the risk of diabetes. The only statements from the Growth Hormone Research Society are that “fasting insulin levels are not routinely measured” and “caution should be exercised when considering the decision of continuing GHT in conditions where there is a known risk of diabetes in the transition age” (88). Also a recent GH safety workshop position paper did not indicate any specific recommendations regarding diabetes risk monitoring in GHD children (89). In our opinion, in agreement with Carel et al. (90), particular attention to glucose metabolism is warranted in such patients and especially in children with already known risk factors for diabetes, both before and at least annually, including an OGTT, which is useful for evaluating any changes in both glucose and insulin levels.

The different methods to study glucose metabolism, the heterogeneity of the populations evaluated, the different doses of GH used together with the variable duration of follow-up may be responsible for discrepancy in the results. Long-term longitudinal studies having glucose homeostasis as their primary outcome are still needed in order to better clarify the real metabolic impact of GHD and GHT in children.

## Author Contributions

AC and CG contributed equally to this work.

## Conflict of Interest Statement

The authors declare that there is no conflict of interest that could be perceived as prejudicing the impartiality of the manuscript.
